# American Psychological Literature

**Published:** 1855-07-01

**Authors:** 


### Art. V.—AMERICAN PSYCHOLOGICAL LITERATURE.

A Pressure of domestic intelligence, relative to the special subject of this journal, has alone prevented us from bringing prominently before our readers the various papers that have appeared from time to time in our able transatlantic contemporary, the *American Journal of Insanity*. We always read this periodical with much pleasure and satisfaction. It is conducted in a liberal, humane, enlightened, and philosophic spirit, free from all those narrow and restricted views that so frequently destroy the practical operation of all psychological inquiries. The editors and contributors to the *American Journal of Insanity* are physicians immediately connected with asylums, and practically conversant with the treatment of the insane. This gives great force and value to their writings. In the recent numbers of the journal, Dr. Kirkbride has been publishing a series of important papers on the "Organization of Hospitals for the Insane," replete with valuable suggestive matter. These papers have appeared in a separate volume, which will be reviewed on another occasion. Dr. Kirkbride writes like a man who has made good use of his eyes. It is said, that "*to see* is not always to *observe*:" but Dr. Kirkbride demonstrates to us that he is able to exercise both powers, and that his faculty of observation is as keen as his sense of sight. We particularly call the attention of all connected with the management of our institutions, both public and private, to this valuable series of papers. The memoir of Dr. Bell, in the October number, is written in a kind and Christian spirit, and, as the record of the life of a man who devoted his best days in promoting the interest of the insane, it is well worthy of perusal. Dr. Galt's "Essays on Insanity in Italy" are highly valuable. The proceedings of the "Association of Medical Officers of American Hospitals for the Insane" are fully reported in various numbers of this journal, and are deeply interesting. We cannot allow this opportunity to escape without thanking the editors of the *American Journal of Insanity* for the kind and generous way in which they invariably have spoken of our humble labours to promote the advancement of psychological literature. The last number of this journal contains a review of Dr. Tuke's prize essay on the "Moral Management of the Insane." The writer of the criticism makes the following sensible remarks on the subject of "Mechanical Restraint in the Treatment of the Insane:"

"Dr. Tuke, after calling to mind the management of the insane prior to 1792, traces the progress of that reform which was commenced simultaneously at that period by Pinel in France and his own progenitor, William Tuke, in England. He views with great satisfaction the prevailing disuse of restraint, and though he does not distinctly favour its entire abolition, yet he intimates that, by the aid of certain practicable substitutes, it may be almost, if not altogether, dispensed with. He pretends to no original ideas on the subject, nor does he go into a very elaborate examination of the merits of the question. This we rather regret, because the friends of the non-restraint practice must now be able, if ever, to meet the objections which have been offered against it. That they never have been fairly disposed of, we firmly believe. On the contrary, if there is any one fact in the management of the insane better settled than any other, we are convinced that it is this—that there are cases of insanity, more or less frequent, in which the highest welfare of the patient is promoted by mechanical restraint.

"Considering how little restraint has been used, for many years, in the principal English establishments, we cannot help thinking that the importance of this question has been greatly over-rated. If the superintendent of a hospital has reason to think that a case occasionally occurs—one or two in a hundred—which is benefited by mechanical restraint, why should he not be allowed to use it? Why should uniformity be required in the matter more than in any point of treatment? If he may be allowed to use narcotics, for instance, or cold baths, or hot baths, to an unprecedented degree, and be praised, perhaps, for his boldness, it is not very obvious why he should be denounced, or regarded as behind the age, because in a few cases he approves of confining his patient's limbs with a bit of leather. If, in 1815, when the monstrous abuses of the English hospitals were brought to light, the cry of 'No restraint' had been raised, it would have been abundantly justified. But it was just at the time when the spirit of improvement had reduced the amount of mechanical restraint to almost nothing—when, in short, this remarkable reform might be safely left to take care of itself—that the public was agitated with this controversy about non-restraint. In the Lincoln Asylum it seems that, from 1829 to 1837, the amount of restraint steadily diminished from thirty-nine, the number of patients being seventy-two, to two, while the number of patients had risen to 130. And yet, in the face of this experience, it was resolved, in the last-mentioned year, to abolish the use of mechanical restraint in every case whatever. A similar piece of history, we presume, would be furnished by many other establishments. We have always supposed that in England the hostility to restraint arose from the fact that in their very large establishments it was quite impossible for the physicians to regulate the application of restraint by their own knowledge of the exigencies of the case, and thus prevent it from becoming, in the hands of attendants, an intolerable evil. Some of the distinguished advocates of non-restraint, we are aware, place themselves upon higher ground than practical expediency. They oppose restraint because, they say, it is never necessary, and always injurious. This conclusion, however, appears to be more like an extravagant expression of warm and earnest feeling, than the result of careful experiment or extensive observations.

"The manner in which this subject has been forced upon the public notice has led, we fear, to another kind of restraint more to be deplored than any that was ever placed on the limbs of the insane. When conversing on this question with the superintendents of hospitals, while in England, a few years ago, we thought we sometimes perceived a fear of maintaining individual convictions against a public sentiment which had become intolerant and proscriptive. When a vexed question has a popular side to it, there is no longer freedom of opinion, nor real progress; because, rather than incur the popular odium, a man will be apt to keep his opinions to himself, instead of permitting them to shape his own practice, and, as far as they deserve, the practice of others. We have no hesitation in saying that the state of feeling and thinking on this subject of restraint, in England, is not that, exactly, best calculated to advance the interests of science or humanity.

"Dr. Tuke *burns*, as we say of children playing at *hide and seek*, when he declares that the non-restraint system can never become practicable nor beneficial, unless the government of the asylum is of a very high moral character. If the character of the management is so effectual in preventing the incidental evils of non-restraint—in making it, as he says, a blessing instead of a curse—it would seem to be an easy inference that it would be equally effectual in preventing the abuses of restraint. So that, in fact, as it respects the welfare of the insane, the really important issue is not between restraint and non-restraint, but between a government which is actuated by high moral considerations, using every available means to promote the good of the patient, and by kindness and vigilance averting every unnecessary abuse, and one careless and indolent, swayed by one idea, and anxious only to catch the popular breeze.

"In this country, fortunately, the question of restraint or non-restraint has always been viewed as one of subordinate importance. We seldom hear it spoken of; and in the meetings of the Association of Superintendents of Hospitals it has never, to our recollection, been a subject of discussion. And yet, we apprehend, it is not often used to an unnecessary extent, even in those hospitals which are most poorly endowed with what Dr. Tuke regards as indispensable substitutes for restraint. It seems to be understood among those who are devoted to this department of the profession, that every one must judge for himself whether the amount of restraint shall be reduced to one per cent. or to zero, and that his conclusion on this point, whatever it may be, cannot fairly subject him to censure. Here, as well as everywhere else, the privilege of free and independent inquiry cannot be invaded without ultimate injury to the cause. If the time should ever come when the superintendents of our hospitals will be obliged to enter upon their duties with the details of their management all prepared for them, seeing everything with the eyes of others, and governed by popular sentiment rather than the sense of right, that time would witness the end of all genuine progress. Let us beware how we allow the first step to be taken in this direction, and resist every attempt to prescribe opinions and practices which should flow only from one's own honest and deliberate convictions."

In noticing the two medico-juridical trials, reported in the October number of the "Psychological Journal," *viz.*, the will case, *Robarts* v. *Kerslake*, and the criminal case of *Mrs. Brough*, the editor expresses his concurrence in the medico-legal view taken by the editor of this journal of these two important cases. This cannot be otherwise than highly gratifying to our feelings. Of the former case, the editor observes, after detailing the salient points given in evidence,

"A careful perusal of all the testimony leads to the conclusion that Mr. Roberts was insane when he executed his will. The act itself, right and proper as it may have been, does not argue that the mind of the testator was sound."

In reference to the case of Mrs. Brough, the editor remarks,—

"We have given all the important testimony in this case, which Dr. Winslow justly remarks, is destined, 'from its peculiar features, to take a permanent position among the *causes célèbres* of British criminal jurisprudence.' The acquittal of the prisoner on the plea of insanity is a recognition of a form of mental disease—or, to speak more correctly, a phase of mental disease—which has usually been regarded by English and American courts as simply the exhibition of ungoverned passion, the consequences of which, if injurious to others, should subject the individual to punishment. Every one at all familiar with the insane knows the power of the passions and impulses over the actions, when the self-conscious, self-governing principle is impaired or suspended. In the case of Mrs. Brough we have a mother who has always been kind and indulgent to her children, and had just nursed them through a long illness. She had previously suffered from cerebral disease and paralysis. She is detected by her husband in what he believes to be a criminal intimacy, and he at once leaves her. Now, here is a great moral shock — a sufficient cause for the sudden development of a paroxysm of mania in a person whose brain was already diseased. But it is said that the act was prompted by revenge,—that she had been detected in infidelity to her marriage vow, and fearing that her children would be taken from her, and that she would be thrown, an outcast from society, upon the world's cold charities, she deliberately and with malice committed the horrid deed. The history of the case, however, precludes such an opinion, and we are pleased to see a decision founded alike upon justice and humanity."

